# Atypical Neurofibroma and Osteosclerotic Metastasis

**DOI:** 10.1155/2012/301437

**Published:** 2012-01-15

**Authors:** Neeraj Varyani, Anubhav Thukral, Sunny Garg, Kailash Kumar Gupta, Ravi Tandon, Kamlakar Tripathi

**Affiliations:** Department of General Medicine, Institute of Medical Sciences, Banaras Hindu University, Uttar Pradesh, Varanasi 221005, India

## Abstract

35-year-old male presented with multiple swellings in left leg, headache, weakness of limbs for 4 months, and blurring of vision for the last 15 days. On examination, he was pale, cachexic with generalized lymphadenopathy and lower motor neuron type weakness of limbs sparing right upper limb. Blood investigations showed anemia with high alkaline phosphatase. Chest radiograph revealed osteosclerotic metastatic lesion in humerus. Biopsy of leg lesion revealed atypical neurofibroma. Computed tomography (CT) of thorax revealed osteoblastic metastasis. Bone marrow aspiration showed cells with round to oval nuclei, fine granular chromatin with large central prominent nucleoli and eosinophilic cytoplasm with acini formation. Magnetic resonance imaging (MRI) of brain and spinal cord defined metastatic leptomeningeal deposits. Cerebrospinal fluid (CSF) cytology was positive for malignant cells. Gastroscopy showed an ulceroinfiltrative growth from stomach which on histopathology revealed diffuse adenocarcinoma. Palliative treatment was given with intrathecal methotrexate and systemic corticosteroid with chemotherapy. Patient's symptom improved drastically, but we lost him to followup.

## 1. Introduction

Gastric carcinoma is the 3rd most common gastrointestinal malignancy after colon and pancreatic carcinoma and is a major cause of morbidity and mortality in many countries of the world. Since stomach is not accessible to clinical examination, gastric carcinoma is a diagnosis often missed by physicians due to varied presentations. Carcinoma stomach manifesting itself with leptomeningeal metastasis and osteosclerotic metastasis is very rare, and presentation in the 4th decade of life is even rarer. This makes our case even more important. So, in a case of osteosclerotic metastasis, gastric carcinoma as a primary should also be kept at the back of our minds. High degree of suspicion is needed to diagnose gastric carcinoma. This fact helped us to diagnose the condition and immediately plan the palliative therapy. 

## 2. Case Presentation

**Figure 1:**
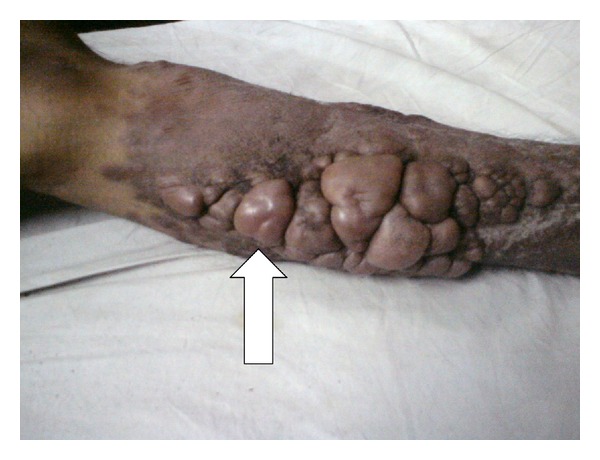
Clinical photograph of the patient showing multiple atypical neurofibromas of left lower limb (shown by arrow).

On October, 2010, a 35-year-old male presented to our outpatient department complaining of multiple swellings on left calf, headache, and weakness of left upper limb followed by both lower limb for the past 4 months. He complained of blurring of vision for last 15 days, but there was no diplopia. Patient developed gradually progressive swellings over left calf involving the whole of leg below knee joint up to the ankle, with reddish brown discoloration of overlying skin (Figure 1). Swellings were not painful, nonitchy with no discharging sinus or ulceration. He denied history of fever, tinnitus and vertigo, or hearing impairment. There was a positive history of previous treatment for pulmonary tuberculosis 4 years back. Patient was an alcoholic and heavy smoker for the past 15 years. 

**Figure 2:**
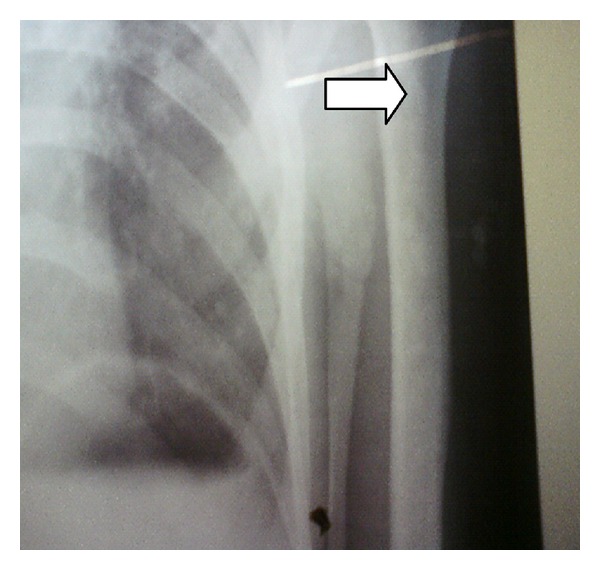
Chest radiograph showing osteosclerotic metastatic lesions in the upper end of humerus (white arrow).

On examination, our patient was severely pale and cachexic with stable vitals. Generalized lymphadenopathy was present. Fundus examination revealed bilateral papilledema. Multiple, nodular, rubbery, and firm swellings of varying sizes ranging from two to seven centimeters (in greatest dimension) were seen conglomerated over the posteromedial aspect of left leg, highly suggestive of neurofibromas. Other neurocutaneous markers for neurofibromatosis such as café-au-lait spots, shagreen's patch, and ash-leaf macules were absent. Slit-lamp examination did not reveal lisch nodules or posterior subcapsular cataract. Nervous system examination revealed positive neck rigidity and Kernig's sign. On further survey, lower motor neuron type of weakness was present in left upper limb and bilateral lower limbs with sparing of right upper limb. Areflexia and impaired vibration and position sense was present in involved limbs. Cranial nerves, autonomic, and cerebellar function tests were normal, and bilateral plantar response was flexor. Chest examination revealed trachea to be deviated to left with signs suggestive of volume loss in left apical lobe. Other systemic examinations revealed no abnormality. Blood investigations revealed anemia (62 gm/L), high alkaline phosphatase (3506 IU/L), and serum PSA of 1.51 mcg/L. Chest radiograph revealed old healed pulmonary tuberculosis of left upper lobe with osteosclerotic metastasis to humerus (Figure 2).

**Figure 3:**
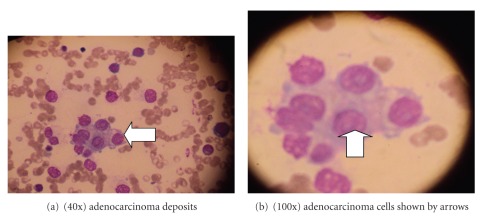
Bone marrow aspirate showing cells with round to oval nuclei and abundant pale eosinophilic and vacuolated cytoplasm.

 Initially, we were mislead by the lesions in the leg considering it to be the primary, but histopathological evaluation of the biopsy specimen revealed atypical neurofibroma. When subjected to immunohistochemical studies, the specimen stained positive for S-100 but negative for calretinin, confirming the diagnosis of neurofibroma. Sonography of abdomen was normal. CT of thorax revealed diffuse, osteoblastic metastasis to sternum, vertebrae, ribs, and humerus. Bone marrow aspirate revealed cells with round to oval nuclei, fine granular chromatin with single large central prominent nucleoli and abundant pale eosinophilic to vacuolated cytoplasm with acini formation suggestive of adenocarcinoma (Figures 3(a) and 3(b)).

**Figure 4:**
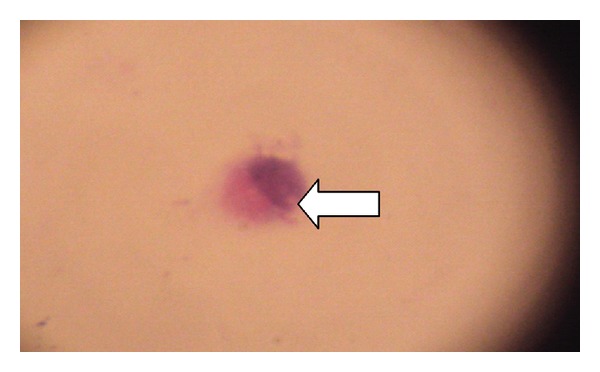
(100x) CSF cytology showing large cells with high nuclear to cytoplasmic ratio. Malignant cell shown by arrow.

MRI of brain and spinal cord with contrast revealed diffuse metastatic deposits over leptomeninges in the brain and spinal cord causing nerve root compression. CSF examination for malignant cytology showed large cells with high nucleus to cytoplasm ratio suggestive of malignant cells (Figure 4). 

**Figure 5:**
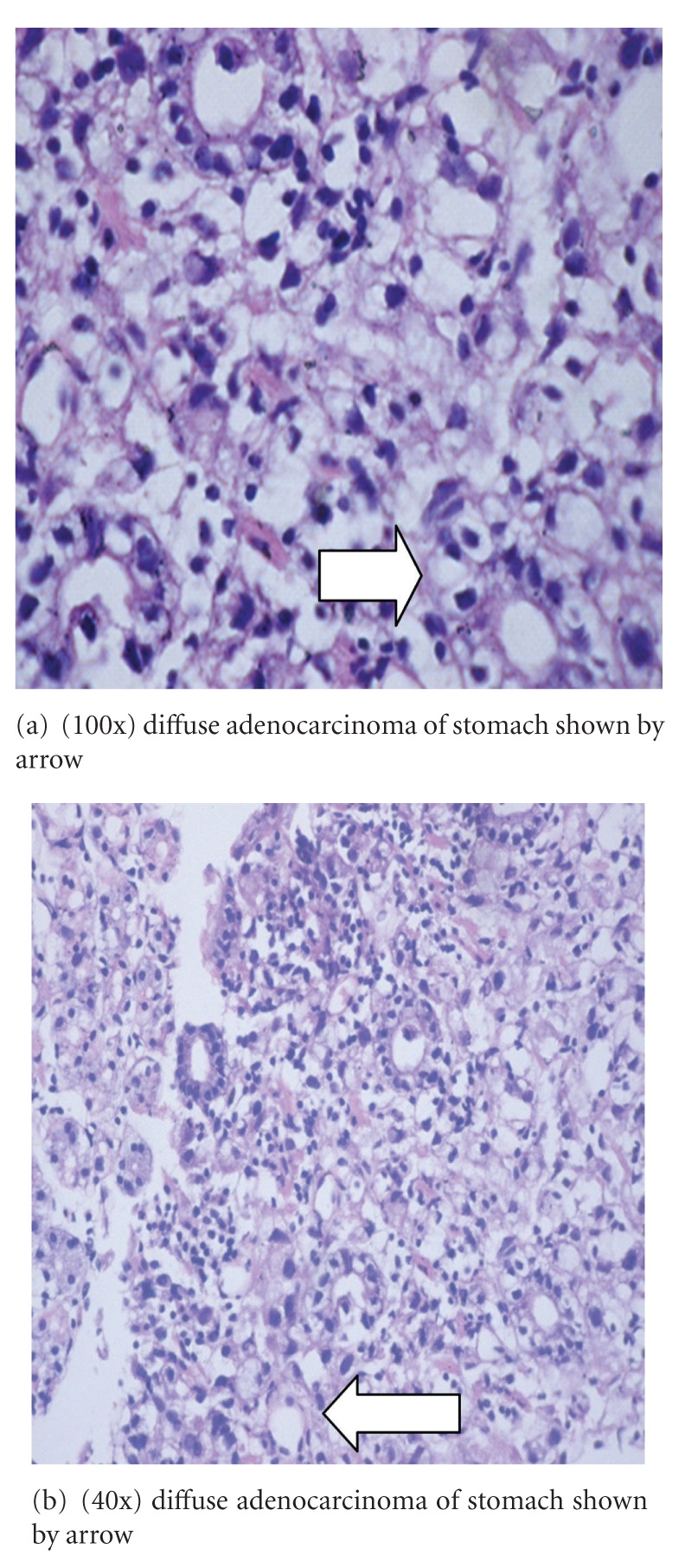
Histopathological examination of ulceroinfiltrative growth of stomach showing malignant signet ring cell and significant dysplasia and altered gland morphology.

Gastroscopy was conducted which showed an ulceroinfiltrative growth from cardia involving the whole of corpus which on biopsy revealed diffuse adenocarcinoma stomach. Histopathological study of biopsy specimen of the growth revealed poorly differentiated adenocarcinoma with architectural disarray and signet-ring cells (Figures 5(a) and 5(b)) which are characteristic finding of diffuse variety of adenocarcinoma stomach.

We planned palliative management for our patient due to his poor Karnofsky's performance status of 10. Treatment was given with intrathecal methotrexate at a dose of 10 mg/m^2^ twice weekly, systemic corticosteroid as intravenous dexamethasone at a dose of 4 mg every 8 hourly, systemic chemotherapy using 5-FU at a dose of 200 mg/m^2^ as intravenous infusion weekly for 6 weeks, folinic acid at a dose of 500 mg/m^2^ as intravenous infusion weekly for 6 weeks 2 hours prior to 5-FU, and cisplatin at a dose of 50 mg/m^2^ as infusion twice weekly were given after premedication with pheniramine, hydrocortisone, and ondansetron. 5-FU and cisplatin were not given on the same day. Patient showed significant improvement in his complaints, but we lost the patient to followup.

## 3. Discussion

Gastric carcinoma is the 3rd most common gastrointestinal malignancy after colon and pancreatic carcinoma. There are about 20,000 new cases of carcinoma of stomach in the USA annually. The incidence has dropped to one-third to what it was 35 years ago. This reflects changes in the prevalence of *H. pylori *infection which has a known etiological role in this disease. The present incidence in American males is ten new cases per 100,000 population per year. Stomach carcinoma is twice as common in males as in women. Almost 95% of gastric carcinoma is adenocarcinoma. Squamous cell tumors of the proximal stomach involve the stomach secondarily from the esophagus. Incidence of bone metastasis in patients of gastric carcinoma was found to be 13.4% in an autopsy study from Japan by Chung et al. [1]. More than 80% of bone metastasis from gastric carcinoma is from poorly differentiated adenocarcinoma. Thoracic and lumbar vertebrae are most frequent sites of bone metastasis, although there are few reported cases of deposits in calcaneum, pelvis, and skull base. Prognosis of patient with bone metastasis is poor with mean survival of less than 5 months. Osteoblastic metastasis is more commonly caused by prostate, breast, and lung together accounting 80% of such metastasis; thyroid, urinary bladder, renal, and stomach are other primaries as shown in the extensive work by Mundy [2]. It is very rare for gastric carcinoma to have osteoblastic metastasis as primary manifestation; most of the metastatic lesions to bone in gastric carcinoma are of osteolytic variety. 6.25% of cases of occult primary with osteoblastic metastasis have gastric carcinoma as shown by Katagiri et al. [3]. Carcinomatous meningitis can present with signs and symptoms at multiple levels of nervous system.

Leptomeningeal metastasis is most commonly caused by carcinoma of breast, lung and melanoma. Incidence of leptomeningeal metastasis in gastric carcinoma is 0.16% with mean survival of 6 to 12 weeks as shown by Anagnostopoulos et al. [4].

Neurofibromas are the most common benign peripheral nerve tumors. Neurofibromas can be further divided into solitary, diffuse, and plexiform varieties. Histopathology classically reveals axons that are incorporated within the tumor mass along with Schwann cells, collagen tissue, perineural cells, and fibroblasts. As the axons are intermixed within the tumor, the tumor cannot be excised without sacrificing a portion of the involved nerve. Diffuse and plexiform varieties are less common than the solitary form. Neurofibromas are commonly found in patients with neurofibromatosis type 1 (NF 1 or von Recklinghausen disease).

 As in our case, there was no family history of similar skin lesions; also there were no signs suggesting neurofibromatosis even after through search in examination and imaging investigations, so we deferred consideration of neurofibromatosis in our case, and molecular search for the neurofibromatosis gene 1 and neurofibromatosis gene 2 was not conducted in our study. Moreover, we never considered gastric adenocarcinoma to be part of the neurocutaneous syndrome.

## 4. Conclusion

This case foretells us that since stomach is not accessible to clinical examination, gastric carcinoma is a diagnosis often missed by physicians due to varied presentations. So, in a case of osteosclerotic metastasis, primary gastric carcinoma should be kept as a differential diagnosis. This fact helped us to diagnose the condition and immediately plan the palliative therapy. Our patient had no locoregional spread to lymph nodes but had bone metastasis. Hematogenous spread to liver was also not seen. Carcinoma stomach manifesting itself with leptomeningeal metastasis and osteosclerotic metastasis is very rare, and presentation in the 4th decade of life is even rarer. This makes our case even more important. Early diagnosis and management of stomach malignancy can help improve patient's quality of life immensely.
